# Robust Optimization of a MEMS Accelerometer Considering Temperature Variations

**DOI:** 10.3390/s150306342

**Published:** 2015-03-16

**Authors:** Guangjun Liu, Feng Yang, Xiaofan Bao, Tao Jiang

**Affiliations:** School of Mechanical Engineering, Tongji University, Shanghai 200092, China; E-Mails: gjliu@126.com (G.L.); 15316800929@163.com (F.Y.); baoxiaofan1122@163.com (X.B.)

**Keywords:** MEMS, accelerometer, robust optimization, temperature variations, sensitivity analysis

## Abstract

A robust optimization approach for a MEMS accelerometer to minimize the effects of temperature variations is presented. The mathematical model of the accelerometer is built. The effects of temperature variations on the output performance of the accelerometer are determined, and thermal deformation of the accelerometer is analyzed. The deviations of the output capacitance and resonance frequency due to temperature fluctuations are calculated and discussed. The sensitivity analysis method is employed to determine the design variables for robust optimization and find out the key structural parameters that have most significant influence on the output capacitance and resonance frequency of the accelerometer. The mathematical model and procedure for the robust optimization of the accelerometer are proposed. The robust optimization problem is solved and discussed. The robust optimization results show that an optimized accelerometer with high sensitivity, high temperature robustness and decoupling structure is finally obtained.

## 1. Introduction

The effect of environmental temperature in MEMS accelerometer structures is more significant than in macro-scaled structures [1,2], as the architecture of a MEMS accelerometer is fabricated with silicon, which is a high temperature-sensitive material and its physical characteristics vary greatly with ambient temperature [3]. The environmental temperature has a great influence upon the performance of a MEMS accelerometer, and errors sources are introduced into the device in its operating environment. The actual performance of the device will deviate from its designed performance when operating in a temperature changing environment. Performance drift due to thermal-mechanical coupling is often found in MEMS. Conventional approaches for MEMS devices calibration rely on third-order thermal models and external temperature sensors, which suffer from the thermal lag and temperature-induced hysteresis [4]. Therefore, new structures with self temperature compensation or robustness to environment are potential alternatives.

Performance shifts caused by temperature variations have become more and more troublesome [5]. To accurately model the behavior of MEMS, it is necessary to accurately compute the coupling [6]. Thermal, structural, and environmental actions should be considered in the modeling, simulation and design of the devices [7]. Multiphysics modeling and simulation are increasingly adopted in revealing and solving coupled multiphysics problems of MEMS [8]. The performance of an accelerometer is concerned with deviations in device performance due to environmental temperature variations. It is important that an accelerometer’s performance is sufficiently robust to temperature variations [9]. An optimal and robust design is highly desirable with regard to device design [10,11]. 

Robust optimization is an engineering methodology for making a product or process insensitivity to the effects of variability so that high-quality products can be produced [12]. A robust design that can reduce the effect of variations from uncertainties is highly necessary to guarantee more reliable performances and improve the yield rate in mass production [13]. A number of robust optimization methods have been applied to design MEMS devices. Robust optimization for MEMS gyroscopes [2,14,15], accelerometer [11], quartz crystal microbalance [12], resonator [16], cantilever [17], micro-mirror [18], filters [19] and magnetometers [20,21] have been reported. These studies concern the robust optimization of MEMS devices considering fabrication errors, and robust optimization on increasing environmental robustness is rarely seen.

In this paper, we present a robust optimization formulation against temperature variations for a MEMS accelerometer. The temperature effects on the performance of the accelerometer are determined. The key parameters that have greatest influence on the accelerometer are found out by sensitivity analysis and therefore the design variables of the robust optimization are selected. The mathematical model considering temperature robustness constraints is built and solved.

## 2. The Accelerometer 

### 2.1. Operating Principle 

**Figure 1:**
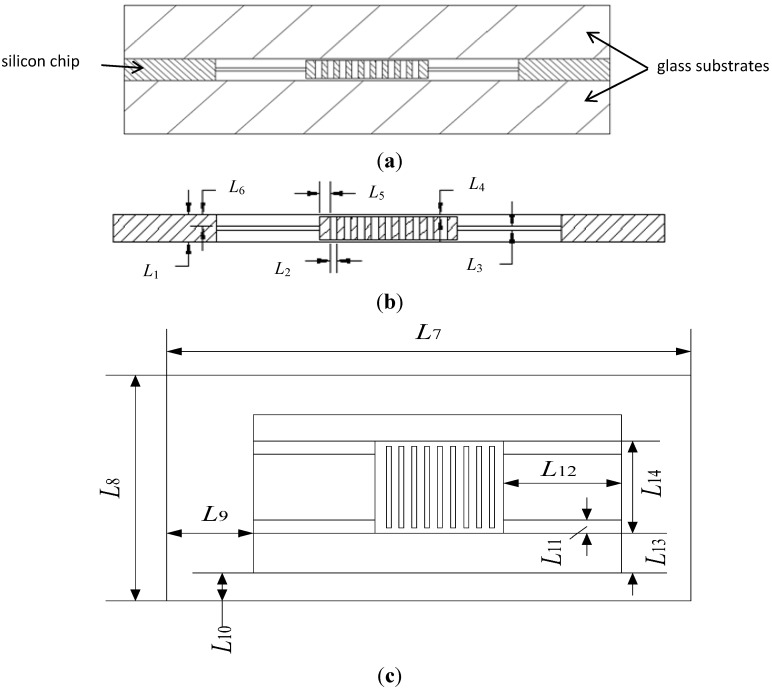
(**a**) Assembly drawing; (**b**) and (**c**) Schematic structure.

The structure of the accelerometer, as shown in Figure 1, is basically a beam-mass structure. The differential capacitance detection is employed to increase output accuracy. The accelerometer has a “sandwich” structure which consists of a silicon chip and two glass substrates, as shown in Figure 1a. The silicon chip placed between the two glass substrates consists of a proof mass a supporting frame and four suspension beams. The proof mass is connected to the supporting frame by the suspension beams. The electrodes on the proof mass and fixed electrodes on glass substrate form the detection capacitors, and there is a detection capacitor on the silicon chip and each glass substrate.

**Figure 2:**
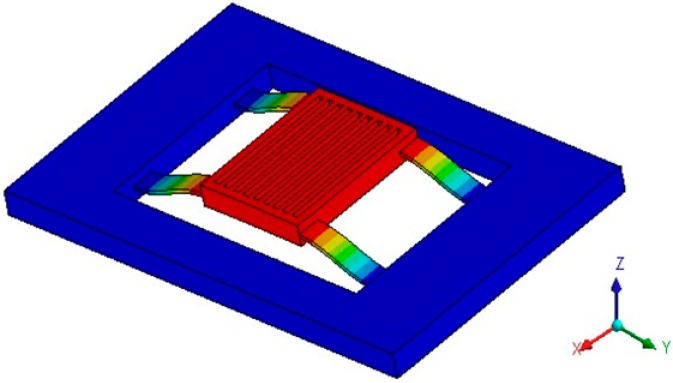
Detection mode of the accelerometer.

The main principle of acceleration detection is that an acceleration applied in the detection direction will cause the proof mass to move from its rest position. The proof mass connected to the supporting frame is free to move in the detection direction (Z-direction). When there is an acceleration applied in Z-direction, the proof mass of the accelerometer will move in the detection direction, and the accelerometer senses a change in electrical capacitance due to the distance change of two capacitors. The resulting capacitance change between the moving electrodes on the proof mass and fixed electrodes can be detected differentially. Accordingly the acceleration is measured. The operating mode (detection mode) is the 1st vibration mode of the accelerometer, as shown in Figure 2.

### 2.2. Calculation of the Output of Detection Capacitance

The basic mathematical model of the accelerometer is a mass-spring-damper system. The input acceleration generates an inertial force to the system. The mechanical model of the accelerometer can be given by the following 2nd-order differential equation:

(1) $$m\ddot{z}+b\dot{z}+kz=ma$$

where *m* is the mass of the proof mass; *z* is the displacement of the proof mass; *b* is the viscous damping coefficient; *k* is the elastic stiffness; *a* is the acceleration applied to the system.

According to the vibration theory, when the accelerometer is in a steady state with constant acceleration input, the displacement of the proof mass tends to a constant:

(2) $$z=\frac{ma}{k}=\frac{a}{\omega_{n}^{2}}$$

where $\omega_{n}$ is the 1st resonance angular frequency of the accelerometer.

When acceleration *a* = 0, the proof mass locates in its equilibrium position, there is no capacitance output. The two differential capacitance $C_{1}$ and $C_{2}$ are:

(3) $$C_{1}=C_{2}=\frac{\varepsilon \varepsilon_{0}A}{d_{0}}=C_{0}$$

where $C_{0}$ is the initial static capacitance of $C_{1}$ and $C_{2}$ ; $\varepsilon_{0}$ is the vacuum dielectric constant; ε is the relative dielectric constant of the medium; A is the area of the capacitive plate; $d_{0}$ is the gap between the moving electrode on the proof mass and fixed electrode on the glass substrate.

When there is an acceleration input, the displacement of the proof mass due to the acceleration will cause capacitance changes of the two capacitors. The capacitance difference of the two capacitors is the output capacitance which is proportional to the displacement. The relationship between the output capacitance ΔC and $C_{0}$ is:

(4) $$\frac{\Delta C}{C_{0}}=2\frac{z}{d_{0}}$$

where $\Delta C=\left|C_{1}-C_{2}\right|$ and $z<<d_{0}$ .

Substituting Equation (2) into Equation (4), the relationship between the output capacitance and input acceleration becomes:

(5) $$\Delta C=\frac{2C_{0}}{d_{0}}\frac{a}{\omega_{n}^{2}}$$

According to Equation (5), the input acceleration is proportional to the output capacitance. The output capacitance can be used to characterize the magnitude of the measured acceleration. 

The sensitivity *K* of output capacitance to the input acceleration is:

(6) $$K=\frac{\Delta C}{a}=\frac{2C_{0}}{d_{0}\omega_{n}^{2}}$$

## 3. Effects of Temperature Variations on the Accelerometer

### 3.1. The Material Properties

When the accelerometer works in a wide temperature range, the temperature variations will result in thermal deformation, thermal stresses and changes in Young’s modulus of the material, and eventually cause output deviations of the accelerometer. As the accelerometer is designed to work in applications where significant temperature variations are expected, the effect of temperature on the device performance is a major concern [22]. Therefore, the effect of thermal-mechanical coupling to the output capacitance needs to be determined for the optimization or temperature compensation of the accelerometer.

The effects of temperature variations on the accelerometer can be decoupled into three different effects: changes in the Young’s modulus, thermal deformation and thermally induced stresses [23].

The influence of temperature on the Young’s modulus of the material changes can be approximated as [24]:

(7) $$E(t)=E(t_{0})+E(t_{0})TC_{E}\Delta t$$

where E(t) is the Young’s modulus under temperature *t*; $E(t_{0})$ is the Young’s modulus under reference temperature $t_{0}$ ; $TC_{E}$ is the temperature coefficient of Young’s modulus; Δt is the change in temperature.

The reference temperature is 20 °C at which the accelerometer is fabricated and packaged. It is supposed that the structure has no thermal deformation at reference temperature. In the operating temperature range of the accelerometer, the thermal expansion coefficient and thermal conductivity of silicon are considered as constants to simplify calculation, as they vary little with the temperature.

### 3.2. Resonance Frequency

**Figure 3:**
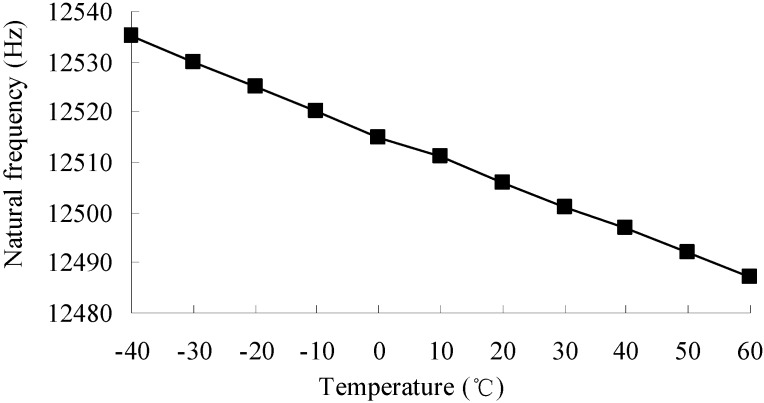
1st resonance frequency *vs.* temperature.

As the volume of the accelerometer is very small and the material (silicon) of the accelerometer has a very high coefficient of thermal conductivity, it is assumed that the temperature of the accelerometer equals the environmental temperature. The accelerometer is assumed to be an elastic body in its working temperature −40–60 °C. Then inertial loads and uniform temperature loads are applied to the finite element model of the accelerometer and a modal analysis and thermal-mechanical coupling analysis are performed. The change of the 1st resonance frequency with temperature is shown in Figure 3.

The 1st mode is the detection mode of the accelerometer. It is seen that the 1st resonance frequency is inversely proportional to the temperature, and the variation of the resonance frequency with temperature is almost linear. The changes in the resonance frequency are caused by the changes in Young’s modulus. As the Young’s modulus changes with temperature linearly, so does the resonance frequency.

### 3.3. Thermal Deformation

Temperature variations could cause thermal deformation of the accelerometer. The acceleration detection of the accelerometer is realized through elastic deformation, so the thermal deformation may influence the output of the accelerometer. 

**Figure 4:**
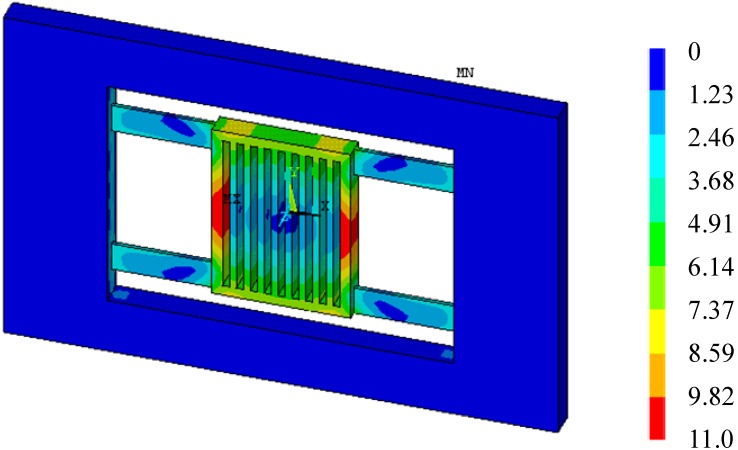
Deformation when temperature arising 20 °C (×10^−8^ m, *a* = 0).

Figure 4 shows the total deformation of the accelerometer with temperature arising from 20 °C to 40 °C when *a* = 0. In fact, Figure 4 is the thermal deformation of the accelerometer, as there is no acceleration input. It is seen that the thermal deformation symmetrical and non-uniform. The edge of the proof mass has the maximum deformation 0.11 µm. The suspension beams also has a relative large deformation up to 0.037 µm. The flatness of the upper and lower surfaces of the proof mass will be affected. The non-uniform thermal deformation of the proof mass means a shape change of the detection capacitors, and will eventually influence the output capacitance.

**Figure 5:**
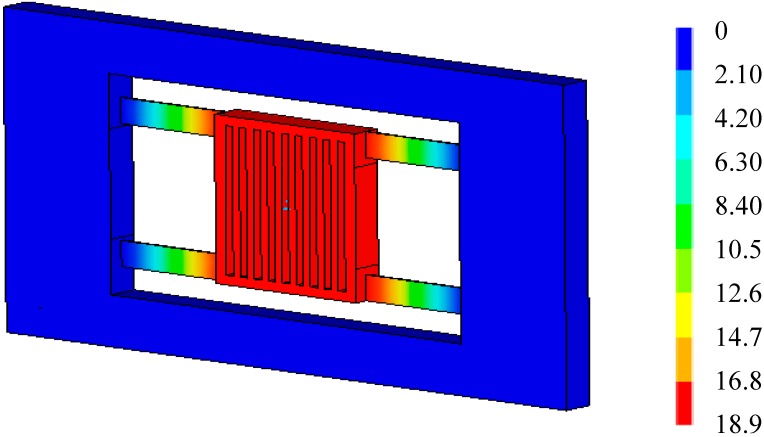
Total deformation at reference temperature 20 °C (×10^−8^, *a* = 50m/s^2^).

**Figure 6:**
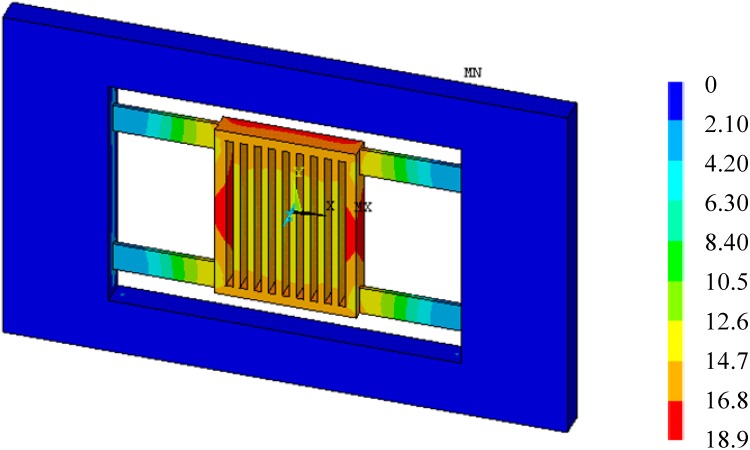
Total deformation at 40 °C (×10^−8^ m, *a* = 50m/s^2^).

After applied inertia loads and temperature loads to the FE model of the accelerometer, the total deformation of the accelerometer at 20 °C (reference temperature) and 40 °C when *a* = 50 m/s^2^ can be obtained, as shown in Figure 5 and Figure 6, respectively. From Figure 5, it is seen that the deformation of the proof mass is uniform, and the deformation is caused by acceleration. From Figure 6, it is seen that the deformation of the proof mass is non-uniform, and the total deformation is a superposition of thermal deformation and mechanical deformation. Though the accelerometer’s deformation at 20 °C and 40 °C both has the maximum deformation 0.189 µm, there will be an output deviation due to the non-uniform deformation at 40 °C.

### 3.4. Output Capacitance

**Figure 7:**
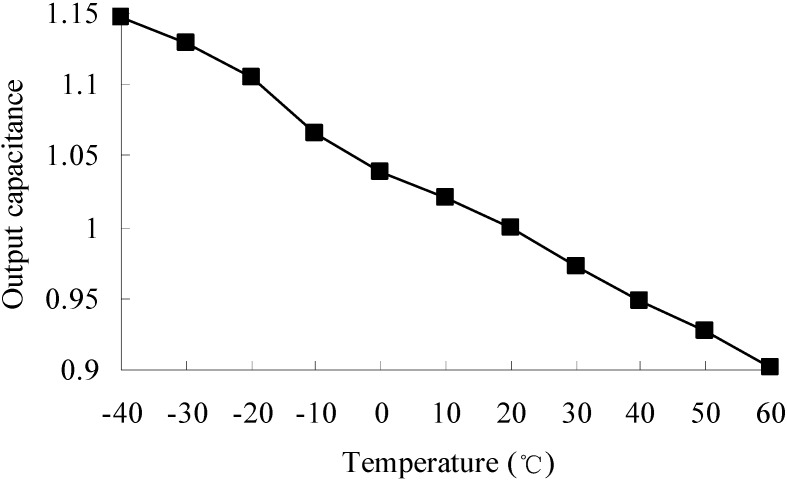
Output capacitance at different temperatures.

Temperature variations result in output deviation due to thermal deformation and changes in material properties. The output capacitance of the accelerometer at different temperatures is shown in Figure 7. The output capacitance at reference temperature 20 °C is set to a unitary value, and the output capacitance at other temperatures is a relative value to the one at 20 °C. 

It is seen that from −40 °C to 60 °C, the output capacitance has a linear change trend. The output capacitance decreases with the increasing of temperature. The maximum output deviation can be 14.7%, which is found at −40 °C. The output deviation is mainly caused by thermal deformation and changes in Young’s modulus.

The temperature variations cause in the performance drift of the accelerometer, and eventually lead to measurement errors. Therefore, considering the environmental temperature variations and temperature-induced thermal-mechanical coupling is necessary in the design of an accelerometer. Temperature compensation method or a robust structure considering uncertainties for the design could be developed [2,25]. Figure 7 provides a reference for temperature compensation and a design objective for robust design by minimizing the influence of temperature variations in relation to the output performance of the accelerometer.

## 4. Sensitivity Analysis of Output Capacitance and Resonance Frequency

### 4.1. Sensitivity Analysis of Output Capacitance and Resonance Frequency to Structural Parameters

Nowadays, many complex MEMS devices are designed and fabricated thanks to the advances in microfabrication techniques [26]. A proper optimization process of MEMS devices is challenged by the increase in design complexity and the coupling in multiple physical domains [27]. The analysis and optimization of a MEMS device is computationally expensive. A set of factors contributes to the computational cost. Therefore, saving computational cost has become a major concern in the optimization and simulation of MEMS devices.

As shown in Figure 1, the accelerometer has 14 independent structural parameters (*L*_1_, *L*_2_, …, *L*_14_) aside from the number of bar electrodes on the proof mass. As the optimization of the accelerometer is a nonlinear problem including thermal-mechanical coupling and may be computationally expensive, it is time-saving to reduce the dimension of the design space and increase the calculation speed. Also, it is important to find out key parameters that determine the performance of and what level of accuracy of a structural parameter is necessary, which can provide a reference for the fabrication processes.

As it is extremely difficult to measure and obtain real statistical distribution of an accelerometer in the real world, sensitivity analysis is often used to determine how “sensitive” a model is to changes in the value of the parameters of the model [28]. Sensitivity analysis of the output capacitance to structural parameters can find out key parameters that have greater influence on the output performance of the accelerometer.

The sensitivity of the output capacitance to a structural parameter $L_{i}$ is defined as:

(8) $$S_{c}=\frac{\left|\Delta C^{\prime }-\Delta C\right|}{\Delta C}$$

where $\Delta C^{\prime }$ is the output capacitance under ΔL .

The initial computational conditions and parameters of the accelerometer are shown in Table 1. The procedure of the sensitivity analysis of output capacitance to variation of structural parameters is as follows:

(1) Calculate the output capacitance of the initial structure;
(2) Change one of the structural parameters with $\Delta L_{i}$ ( $\Delta L_{i}/L_{i}=1\%$ ), and keep other parameters unchanged;
(3) Calculate the sensitivity of the output capacitance to the parameter according to Equation (8);
(4) Find out the key parameters that have greatest influence on the output capacitance.

**Table 1:** Computational conditions of the accelerometer.

Computational conditions	Value
Initial nodal displacements	0
Initial nodal velocities	0
Acceleration	50 m/s^2^
Young’s modulus	1.7×10^5^ MPa
Poisson’s ratio	0.3
Density	2330 kg/m^3^
Temperature	20 °C

The sensitivity analysis of the resonance frequency $S_{N}$ is defined as:

(9) $$S_{N}=\frac{\left|\omega^{\prime }-\omega \right|}{\omega }$$

where ω is the 1st resonance frequency of the accelerometer; $\omega^{\prime }$ is the 1st resonance frequency of the accelerometer under ΔL . The procedure is similar to the one of output capacitance.

### 4.2. Results of Sensitivity Analysis

The results of sensitivity analysis are shown in Table 2. It is found that $L_{7}$ , $L_{8}$ , $L_{9}$ , $L_{10}$ , $L_{11}$ and $L_{12}$ have greater influence on the output capacitance than other parameters, and $L_{7}$ , $L_{8}$ , $L_{10}$ , $L_{11}$ and $L_{12}$ have greater influence on the 1st resonance frequency than other parameters.

**Table 2:** Results of sensitivity analysis.

Parameter	*L*_1_	*L*_2_	*L*_3_	*L*_4_	*L*_5_	*L*_6_	*L*_6_
*S*_c_ (%)	1.294	1.705	3.197	1.159	3.076	2.118	9.276
*S_N_* (%)	2.011	1.977	2.859	2.898	0.975	1.290	4.612
**Parameter**	***L*_8_**	***L*_9_**	***L*_10_**	***L*_11_**	***L*_12_**	***L*_13_**	***L*_14_**
*S*_c_ (%)	14.477	3.804	4.584	4.419	4.162	2.367	1.098
*S_N_* (%)	11.401	3.172	3.913	3.121	4.166	2.339	1.901

Through the sensitivity analysis, the following conclusions can be obtained:

(1) The six parameters that have greater influence on the output capacitance include the five parameters that have greater influence on the 1st resonance frequency, which shows a relationship between the resonance frequency and output capacitance. This can be explained by Equation (5).
(2) The length $L_{7}$ and width $L_{8}$ of the accelerometer have the greatest influence on the performance.
(3) The structural parameters related to proof mass also have significant influence on the performance.
(4) The length of the suspension beams $L_{12}$ determines the stiffness of the beams, so it will influence the output capacitance and resonance frequency. However, the width $L_{11}$ and height $L_{3}$ of the suspension beams have a relative smaller influence on the performance of the accelerometer.
(5) It is recommend to keep more attention on $L_{7}$ , $L_{8}$ , $L_{9}$ , $L_{10}$ , $L_{11}$ and $L_{12}$ in fabrication process, as they have more influence on the performance of the accelerometer.

The sensitivity of the output capacitance to fabrication variations is calculated under the condition that the percentage coefficients of all structural parameters are supposed to be 1%. It is unnecessary to know the values of percentage (or dispersion) coefficients of stochastic structural parameters in advance [15]. This means robustness can be achieved without any statistical information on the uncertainties.

## 5. Robust Optimization of the Accelerometer

### 5.1. Design Variables

According to the results of sensitivity analysis, we adopt the structural parameters $L_{7}$ , $L_{8}$ , $L_{9}$ , $L_{10}$ , $L_{11}$ and $L_{12}$ as the design variables in the robust optimization of the accelerometer as: please confirm the following equation

(10) $$X=[\begin{array}{cccccc} x_{1} & x_{2} & x_{3} & x_{4} & x_{5} & x_{6} \end{array}]$$

### 5.2. Objective Functions

The purpose of the robust optimization is to find a configuration set in which the structural performance is less sensitive to the fluctuations of parameters considering variability, and the designed performance is maximized [29]. 

For the accelerometer, the sensitivity K of output capacitance to the input acceleration should be maximized:

(11) $$\text{Max }f(X)=\frac{K(X)}{K_{0}}$$

where $K_{0}$ is the initial value of sensitivity K .

### 5.3. Constraints Conditions

The constraint conditions of robust optimization include geometrical constraints, vibration modes constraints, and robustness constraints. 

#### 5.3.1. Geometrical Constraints

Based on the structural characteristics of the accelerometer, as shown in Figure 1 and Figure 2, the geometrical constraint conditions are given by the following:

(12) $$L_{\text{min}}\leq x_{i}\leq L_{\text{max}}\;(i=\text{1, 2, …, 6})$$

where $L_{\text{max}}$ and $L_{\text{min}}$ are the upper and lower bounds for $x_{i}$ , respectively.

#### 5.3.2. Vibration Modes Constraints

An important consideration in the accelerometer optimization is that there must be no coupling between the mode of vibration in the detection direction and other vibration modes [1]. The frequency difference $f_{N}$ between the operating mode and its neighbor mode should be greater than the present one to avoid modes coupling:

(13) $$f_{N}\geq \omega_{2}-\omega$$

where $\omega_{2}$ the 2nd resonance frequency of the accelerometer.

#### 5.3.3. Robustness Constraints

It is expected that the temperature variations have minimum influence on the output of the accelerometer. The robust optimization procedure is justified for stochastic systems by noting that the variances of the outputs can be determined from the sensitivities of the inputs if the output function is modeled using a first-order Taylor series, and minimizing the magnitude of the sensitivities to the inputs tends to minimize the variances of the outputs [30]. The 1st resonance frequency ω of the accelerometer is:

(14) $$\omega =\frac{1}{2\pi }\sqrt{\frac{k}{m}}$$

ω is a random variable when considering the influence of temperature. Supposing that ω is a function of a random variable ζ , ω can be approximately extended at ζ=0 according to the Taylor expansion:

(15) $$\omega (\xi )\approx \omega (0)+\xi {\frac{\partial \omega }{\partial \xi }|}_{\zeta =0}$$

Let:

(16) $${\frac{\partial \omega }{\partial \xi }|}_{\zeta =0}=0$$

So the influence of ζ on ω will be reduced from a 1st-order quantity to a second-order one. Then substituting Equation (14) into Equation (16), we can write the following:

(17) $$\frac{\partial \omega }{\partial \xi }=\frac{1}{2\pi }{\left(\frac{k}{m}\right)}^{-\frac{1}{2}}\frac{\frac{\partial k}{\partial \xi }m-\frac{\partial m}{\partial \xi }k}{m^{2}}$$

According to Equation (16), Equation (17) can be simplified as:

(18) $${\left(\frac{\partial k}{\partial \xi }m-\frac{\partial m}{\partial \xi }k\right)|}_{\zeta =0}=0$$

Then Equation (18) can be rewritten as follows:

(18) $${\left(\frac{\partial k}{\partial \xi }/k\right)|}_{\zeta =0}={\left(\frac{\partial m}{\partial \xi }/m\right)|}_{\zeta =0}$$

Equation (19) means that the unit stiffness sensitivity of the spring beams should be equal to the unit mass sensitivity of the proof mass. Equation (19) is the robustness constraints for the robust optimization of the accelerometer, and the corresponding relationship among design variables can be finally obtained. The robustness constraints can be expressed as:

(19) $$h\left(X\right)={\left(\frac{\partial k}{\partial \zeta }/k\right)|}_{\zeta =0}={\left(\frac{\partial m}{\partial \zeta }/m\right)|}_{\zeta =0}\leq \varepsilon$$

where ε is a relative small positive number. 

### 5.4. Procedure of Robust Optimization

**Figure 8:**
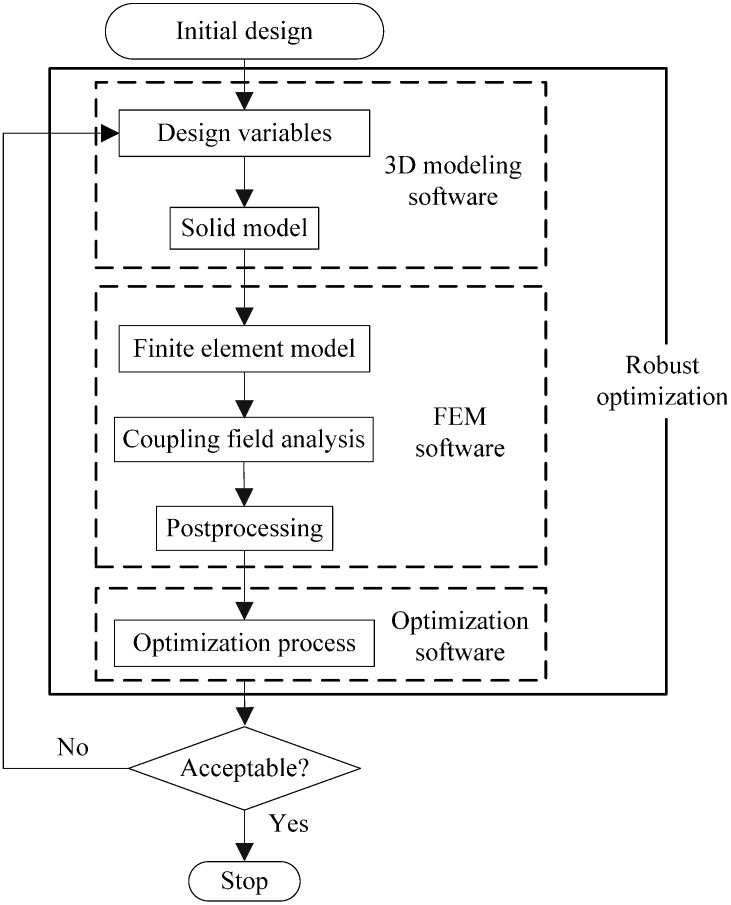
Procedure of robust optimization.

The procedure of the robust optimization for the accelerometer is shown in Figure 8. The robust optimization process is as follows:

(1) Determine design variables according to the results of sensitivity analysis;
(2) Build the solid model of the accelerometer using a 3D modeling software;
(3) Input the solid model into a FEM software, then mesh it;
(4) Apply constraints and loads to the FEM model, and perform FEA;
(5) Calculate output capacitance;
(6) Perform optimization and update design variables;
(7) Check and evaluate the convergence criterion. If the criterion is satisfied, the process is terminated. If not so, go to the Step (2) with updated design variables.

### 5.5. Results of Robust Optimization

**Figure 9:**
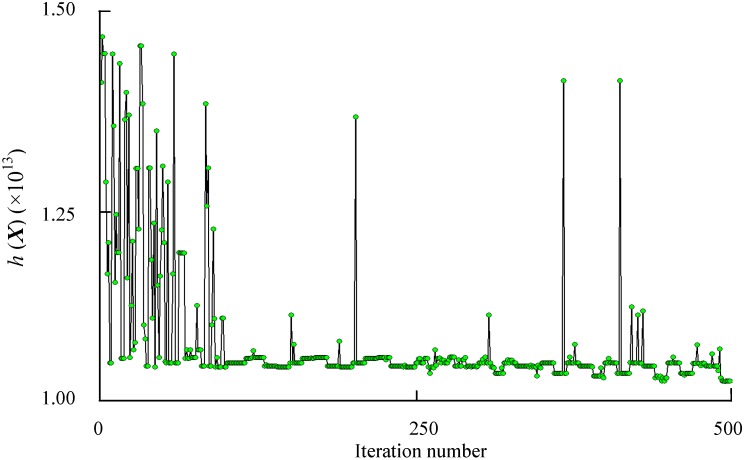
History of robustness constraints h(X) .

**Figure 10:**
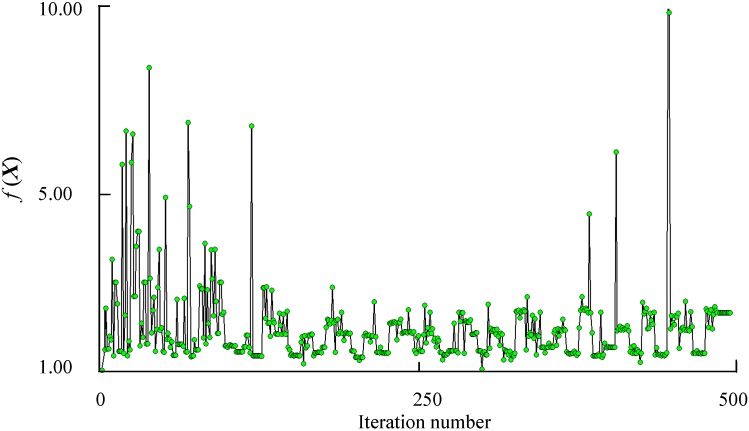
History of objective function f(X) .

The software iSIGHT and Multi-island Genetic Algorithm [31] are employed to solve the optimization problem. The iteration processes of the robustness constraints h(X) and objective function f(X) are shown in Figure 9 and Figure 10, respectively. The optimization results and comparison of initial design and robust optimization are shown in Table 3. 

**Figure 11:**
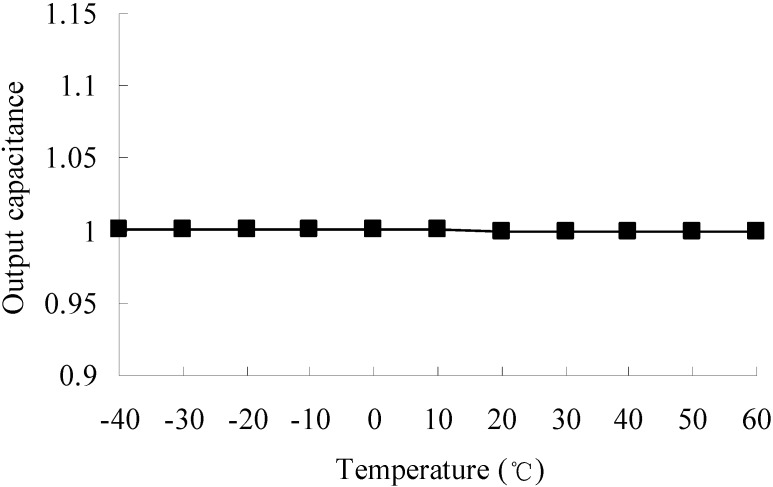
Output capacitance of the optimized accelerometer at different temperatures.

The optimized structure of the accelerometer is built according to Table 3. The optimized parameters are given at an accuracy of sub-nm. However, in modeling of the optimized accelerometer in a 3D modeling software, the input parameters will be reset to the default accuracy of the software automatically. We set 0.0001 mm as the default accuracy of the 3D modeling software Then a calculation of the output capacitance at different temperatures is conducted. The output capacitance at reference temperature 20 °C is set to a unitary value. The output capacitance of the optimized accelerometer at different temperatures is shown in Figure 11.

As shown in Table 3 and Figure 11, a robustness increase to temperature variations is found. The robustness constraints h(X) drops by 28.2%, which means that the performance fluctuation is less than the initial structure and the robust structure has the performance against temperature variations. The sensitivity of output capacitance increases by 244.2%, and the output performance is therefore improved. The frequency difference of the 1st and 2nd resonance frequencies increases by 54.5%. We finally obtain an optimized accelerometer with high sensitivity, high temperature robustness and decoupling structure. The results show that the robust optimization can obtain not only an improved performance but also a higher yield with minimum temperature error information.

The proposed robust optimization formulation is practically applicable since no statistical information on the uncertainties is required during the optimization process in advance and can achieve robustness effectively. Considering that the temperature-induced error is one of the main error sources of the accelerometer, the robust optimization method can be valuable for practical accelerometer’s design.

**Table 3:** Comparison of initial design and robust optimization.

Description	Symbols	Initial Design	Robust Optimization
Design variables (mm)	*x*_1_	8	9.3398106
	*x*_2_	5	5.9304183
	*x*_3_	1.5	1.7980107
	*x*_4_	0.8	0.7820910
	*x*_5_	0.4	0.44435784
	*x*_6_	1.5	1.79393558
1st resonance frequency (Hz)	ω	12727	16180
2nd resonance frequency (Hz)	$\omega_{2}$	19184	26156
Frequency difference (Hz)	*f_N_*	6457	9976
Sensitivity of output capacitance (10^−12^ F/m·s^−2^)	*K*	2.5947	8.9323
Robust constraints	h(X)	1.4356 × 10^13^	1.0314 × 10^13^

## 6. Conclusions

(1) The mathematical model of the MEMS accelerometer is built, and the calculation method of the output capacitance is proposed. The relationship between the input acceleration and output capacitance are determined.
(2) The effects of temperature variations on the accelerometer are investigated. Thermal deformation of the accelerometer is analyzed. When temperature arising from 20 °C to 40 °C, the proof mass has the maximum deformation 0.11 μm, and the suspension beams also has a relative large deformation up to 0.037 µm. The thermal deformation finally leads to output errors of the accelerometer. The deviations of the resonance frequency and output capacitance of the accelerometer due to temperature variations are calculated. It is found that the temperature variations have little influence on the resonance frequency and have a significant influence on the output capacitance. Temperature variations can result in maximum output deviation by 14.7%.
(3) A sensitivity analysis of the output capacitance and resonance frequency of the accelerometer is performed. The structural parameters that have greatest influence on the output capacitance are found out. The length and width of the accelerometer as well as length of the suspension beams are considered the most significant parameters determining the output performance of the accelerometer, which provides a reference for the fabrication process of the accelerometer. According to the results of sensitivity analysis, six structural parameters are selected as design variables for the robust optimization of the accelerometer. The dimensions of design space for the robust optimization are therefore reduced, and a time-saving optimization could be carried out.
(4) The robust optimization procedure of the accelerometer is presented. The mathematical model for the robust optimization is proposed, and an optimized structure robust to temperature variations is obtained. The optimization results show that the sensitivity of output capacitance increases by 244.2%. An optimized accelerometer with high sensitivity, high temperature robustness and decoupling structure is finally obtained.
